# Variation in gene expression profile with aging of *Pinus radiata *D. Don

**DOI:** 10.1186/1753-6561-5-S7-P62

**Published:** 2011-09-13

**Authors:** Carolina Alvarez, Luis Valledor, Rodrigo Hasbún, Manuel Sanchez-Olate, Darcy Ríos

**Affiliations:** 1 Plant Tissue Culture Laboratory, University of Concepción, Chile; 2 Molecular Systems Biology, University of Viena, Austria; 3 Genómica Forestal S.A Biotechnologyy center, University of Concepción, Chile

## 

Phase change in higher plants, from juvenile to mature, affects the reproductive competence, morphology and growth rate, as well as the regenerative potential of tissue explants. However, in conifers these changes can be achieved before the onset of flowering and at the beginning of the seed production. Early maturational changes in conifers are very obvious, severe and overall irreversible [1], and are characterized by lost of morphogenetic capacity. This may affect clonal multiplication programs and genetic manipulation, due mainly to the decline in adventitious rooting capacity which is the most marked maturation and aging effect. Despite the serious effects of these processes, little is known about their basic regulation in forest clonal propagation programs. The latest researches regarding this subject are related to auxins changes during maturation [2], epigenetic variations during phase-change, like DNA methylation [3], and proteomic changes during needle maturation [4]. This work has been performed to gain deeper insight into the genetic mechanisms that regulates the loss of morphogenetic capacity with the aging process in *P. radiata.*

*P. radiata* ortets of different age were brought from La Posada nursery at BioBio region, Chile. Needles were collected in August, frozen in liquid nitrogen and stored at -80°C until its RNA extraction. RNA was isolated from 100 mg of frozen tissue according to Chang [5], the RNA extracts were purified with RNeasy Clean up and treated with RNase-free DNase (both from QIAGEN). Gene expression analysis was done by a reverse dot blot hybridization technique by comparing the transcription levels of 174 genes in 1, 3 and 5 years old ortets using the methodology described by Valledor *et al.*[4]. The 174 genes analyzed were classified into categories being the most important: Chloroplast and photosynthesis, protein translation folding, modification and degradation and proteins involved in transcription and DNA replication. Gene expression was quantified as signal intensity using the Gel Pro-analyzer 3.1.The values were normalized dividing every spot intensity by the average of the intensity of each membrane.

**Figure 1:**
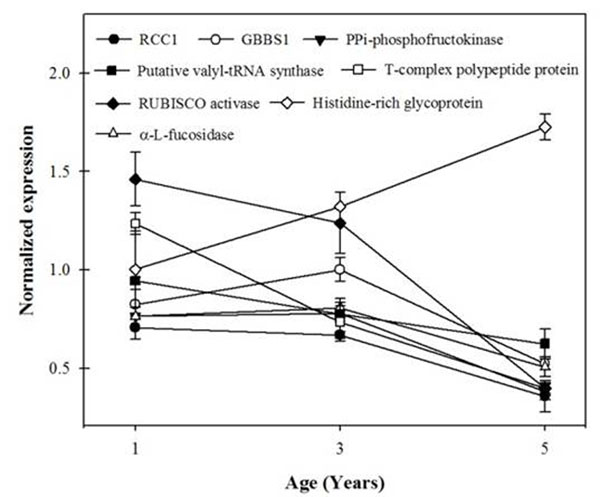
Normalized gene expression of: regulator of chromosome condesnation (RCCI), granule-bond starch synthase (GBBS1), putative valyl-tRNA synthase, T-complex polypeptide protein (TCP-1), RUBISCO activase, histidine-rich glycoprotein and α-L-fuocosidase *Pinus radiata* genes. Values are means ±SE.

From the 174 studied sequences only 56 were differentially expressed among ortets of different ages. From the 56 genes differentially expressed, 27 showed a clear variation tendency with age, from which 21 were down-regulated and 6 were up-regulated, reflecting a higher number of active pathways in the younger ortets. GBBS1, PPi-phosphofructokinase and α-L-fucosidase (Figure 1) genes are related with carbohydrates and carbon metabolism that were affected by the ortet age; all were down-regulated with the increasing age, showing a significantly lower level of transcription in ortets of 5 years old. Both PPi-phosphofructokinase and α-L-fucosidase have been correlated to a higher level of expression in younger and metabolically active tissues. Specifically the *Arabidopsis thaliana* fucosidase (*At*FXG1) has shown a higher expression level in younger leaves than in the older ones, and it has been proposed that it has a role as a growth regulator. The previous results are consistent with the higher expression level in RUBISCO activase (figure 1) in the younger ortets (1 and 3 year old), which in turn agree with different studies, indicating that photosynthesis is reduced with increasing age of shrubs and trees [7] and higher in the younger ones.

Transcription regulated protein family were mainly over expressed in 1 year old ortets. It is the case of the chromosome condensation gene regulator (RCC1) (figure 1), which inhibit the chromosome condensation until G2 phase of the cell cycle and is required for the formation of the mitotic spindle [7].This result indicates that 1 year old ortets are more transcriptionally active. Likewise, proteins like Putative valyl-tRNA synthase and T-complex polypeptide protein like (TCP-1) (figure 1), involved in processes of protein modification and translation, were up-regulated in 1 year old ortets, indicating that juvenile plants are more active at protein synthesis and modification level. In contrast 5 year old ortets presented a down-regulation in these genes expression. Additionally, in 5 year old radiata pines genes like histidine-rich glycoproteins (figure 1) were up-regulated, this type of protein have been found to be a component of the cell wall, like histidine-rich extensin in *Zea mays* [9], this is consistent with the increase in cell wall synthesis in older plants.

Thus, this work has provided an initial insight of the pathways and mechanisms that may be involved in the loss of morphogenetic capacity caused by aging in *P. radiata* ortets. Juvenile ortets showed higher expression in proteins related with transcription activators, photosynthesis and carbon and carbohydrate metabolism, while older *P. radiata* ortets showed a down-regulation in the same genes and a increase in genes related to cell wall synthesis, indicating that these might be candidates genes to be markers of the aging and maturation process, results that will be complemented and validated with qRT-PCR and proteome studies.
